# Multilayered Connective Tissue Grafting Technique to Improve Aesthetics after Failed GBR: A Clinical Case Report of 16 Months Follow-Up

**DOI:** 10.1155/2020/3906468

**Published:** 2020-07-22

**Authors:** Gabriele Villa, Gionata Bellucci, Simone Magnolo, Farah Asa'ad

**Affiliations:** ^1^Unit of Periodontology, Fondazione IRCCS Ca' Granda Ospedale Maggiore Policlinico, University of Milan, Milan, Italy; ^2^Private Practice, Biella, Italy; ^3^Department of Biomaterials, Institute of Clinical Sciences, The Sahlgrenska Academy, University of Gothenburg, Göteborg, Sweden

## Abstract

**Objective:**

This case report describes soft-tissue management after a failed GBR procedure to enhance the soft-tissue quality, quantity, and aesthetic outcomes. *Case Presentation*. A 38-year-old patient visited the Dental Clinic of the Ospedale Maggiore Policlinico, University of Milan, Milan, Italy, for a chief complaint of unsatisfactory aesthetics of the anterior maxillary area. Dental history disclosed failed preimplant vertical bone augmentation with GBR procedure in the area of the left maxillary central incisor resulting in a severe gingival recession of adjacent teeth and compromised soft-tissue quantity and quality and severe ridge atrophy (class III). Multilayered connective tissue grafting technique, in a two-step mucogingival surgery, was used to cover the gingival recessions, reach even gingival margin, and reconstruct the defect. Frenectomy was done after the second mucogingival surgery to relieve the muscle attachment. A definitive cantilever bridge was placed to restore the left and right maxillary central incisors, and a satisfactory aesthetic outcome was reached.

**Conclusions:**

Multilayered connective tissue grafting technique might be successful in correcting soft-tissue quantity and quality in class III ridge defects.

## 1. Introduction

Bone augmentation procedures are routinely applied in cases of alveolar ridge deficiency, in order to achieve sufficient vertical and/or horizontal bone volume, which ensures successful outcomes of dental implant therapy [1]. In general, vertical bone augmentation [2] procedures are more challenging than horizontal bone augmentation procedures and employ various techniques: vertical guided bone regeneration (GBR), onlay grafting, inlay grafting, split bone block technique, and distraction osteogenesis [3–5]. These procedures are frequently associated with high complication rates, reportedly up to 45% [4], such as soft-tissue dehiscence and subsequent exposure of the bone grafting material into the oral cavity [6]. Although most of bone augmentation procedures require autogenous bone and/or bone graft substitutes to achieve successful outcomes of dental implant therapy [5], soft-tissue augmentation might also be necessary to enhance the final outcomes, especially in the anterior maxilla [7].

Management of vertical bone augmentation failures can be quite challenging, not only because of a more pronounced bone deficiency afterwards but also due to compromised quantity and quality of soft tissues at the surgical site and gingival recessions/loss of attachment on adjacent teeth. Therefore, management of bone augmentation failures frequently requires enhancement of soft-tissue quantity and quality through soft-tissue grafting procedures. Specifically, subepithelial connective tissue grafts [8, 9] and deepithelialized free gingival grafts [10] can be utilized for the purposes of increasing soft-tissue volume and covering gingival recessions around adjacent teeth.

In the present case report, we describe for the first time the application of a novel multilayered soft-tissue grafting technique, with multiple connective tissue grafts, obtained by deepithelializing multiple free gingival grafts, to augment a class III Seibert defect [11] in the anterior maxilla that resulted from a previously failed vertical GBR. Since the patient was completely unsatisfied with the poor aesthetic appearance in the anterior maxilla and refused to undergo another bone augmentation procedure, soft-tissue grafting was adopted to correct the vertical GBR failure.

## 2. Case Presentation

In 2017, a 38-year-old Caucasian female visited the Dental Clinic of the Ospedale Maggiore Policlinico, University of Milan, Milan, Italy, with a chief complaint of unsatisfactory aesthetic appearance of the anterior maxillary area.

**Figure 1:**
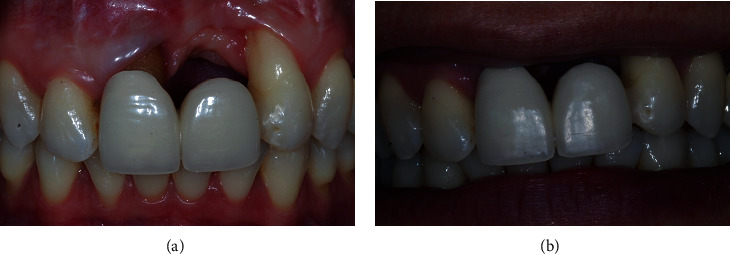
(a, b) Site of chief complaint revealing unsatisfactory aesthetic appearance.

Intraoral examination revealed severe vertical bone loss and scarred soft tissues in the area of the left maxillary central incisor, which was replaced by a pontic (Figures 1(a) and 1(b)).

Severe gingival recession was evident on the left maxillary lateral incisor and the right maxillary central incisor, both of which had degree II mobility.

**Figure 2:**
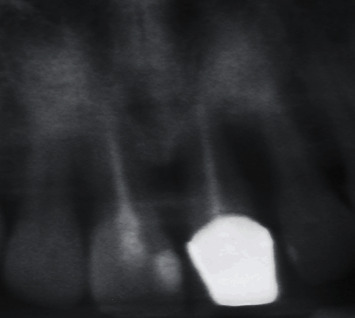
Section from panoramic radiograph representing the site of chief complaint, prior to the attempted extraction of the left maxillary central incisor. Severe vertical bone defect around the left maxillary central incisor is evident.

Dental history revealed that the left maxillary central incisor was extracted due to periodontal disease (Figure 2) and that a preimplant vertical GBR utilizing a titanium-reinforced nonresorbable high-density e-PTFE membrane with autogenous graft/xenograft of a 1 : 1 ratio was performed in the area four months prior to the patient's visit to the university's dental clinic.

The PTFE membrane was exposed and infected, but the patient was away on vacation and could not visit the dentist to manage the case. Therefore, the infected membrane stayed in situ for one month, which resulted in complications in soft-tissue healing, scarred soft tissues in the region of the left maxillary central incisor and severe gingival recessions of the adjacent teeth, resulting in a compromised soft-tissue quantity and quality in the anterior maxillary area. The patient was unsatisfied with the overall aesthetic appearance. The patient did not report any pain in the area of chief complaint. Cone-beam computed tomography (CBCT) revealed a severe vertical bone defect. Due to the ridge defect and lack of soft-tissue quantity, the discrepancy in the location of the gingival margin was about 1 cm (Figure 1(a)).

To manage this case, enhancement of the soft-tissue quantity and quality and improvement of the aesthetic outcomes were planned in a multistep soft-tissue reconstruction surgery.

## 3. Case Management

### 3.1. First Mucogingival Surgery

**Figure 3:**
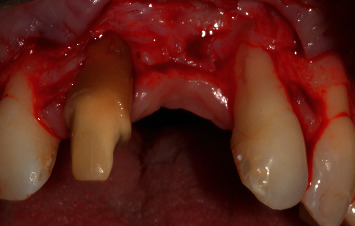
Flap is released (split-full-split) following coronally advanced flap design (as described by Zucchelli & De Sanctis).

The flap design applied to manage this case is presented in Figure 3. The flap design followed the one described by Zucchelli and De Sanctis [12, 13] to manage multiple gingival recessions with coronally advanced flap adding two vertical releasing incisions, one distal to the left maxillary canine and one distal to the right maxillary canine, going about 4-5 mm in the alveolar mucosa. On the alveolar crest, a horizontal incision was done on the vestibular side. All the incisions were split-thickness. Deep split-thickness elevation was done 3-4 mm parallel to the bone. On the palatal side, “palatal island flap” was performed as described by Tinti et al. [14] and modified by Hürzeler et al. [15], in order to achieve primary intention closure. In brief, a horizontal incision was done in the palatal side, connecting the palatal sides of the right maxillary central incisor and the left maxillary lateral incisor, followed by two full thickness releasing vertical incisions that were then connected by a full-thickness horizontal incision, parallel to the first horizontal incision, about 10 mm more apically. At this point, a split-thickness incision was done from the coronal horizontal incision for about 5 mm into the apical direction, and another split-thickness incision was done from the apical horizontal incision for about 3 mm into the coronal direction, all done using a microblade. All root surfaces were dried and conditioned with EDTA for 2 minutes and then with Emdogain.

After the elevation of the flap, the occlusal surface between the two horizontal incisions in the edentulous area was deepithelialized, as it was the recipient site for the soft-tissue graft.

Afterwards, free gingival grafts (FGGs) were harvested from the right and left sides of the palate, from the second molar up to the lateral incisor, as described by Zucchelli et al. [16]. Both grafts were deepithelialized with a 15C blade, to obtain connective tissue grafts.

**Figure 4:**
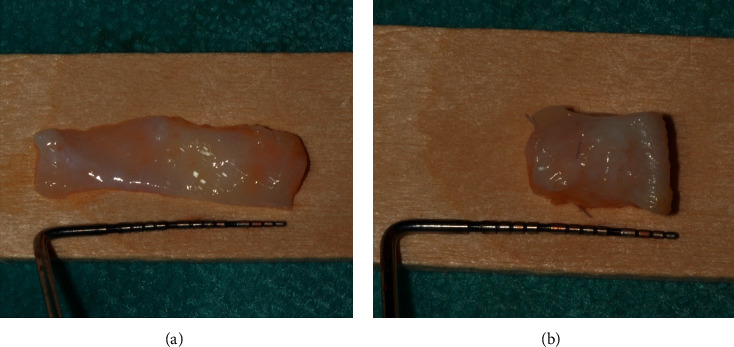
(a) Deepithelialized free gingival graft. (b) Connective tissue graft folded in two layers and then sutured, to gain soft-tissue thickness in the vertical direction.

**Figure 5:**
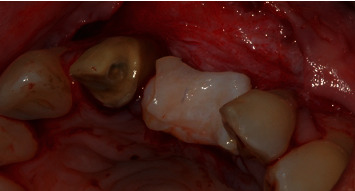
Folded connective tissue graft sutured at the defect site.

**Figure 6:**
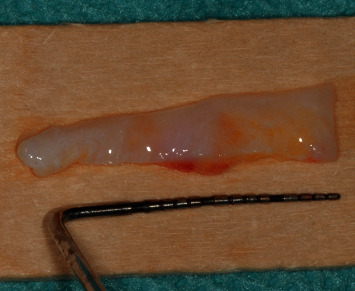
Second deepithelialized free gingival graft.

**Figure 7:**
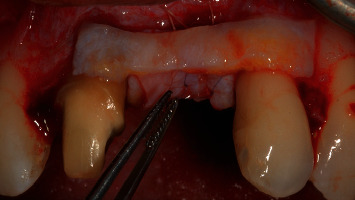
First part of the second deepithelialized free gingival graft sutured on the buccal side.

The first graft, harvested from the right palate, of about 20 mm in length (Figure 4(a)), was folded (Figure 4(b)), and then secured by two vertical mattress sutures, with 7/0 PGA sutures, to form a two-layered graft. The two-layered graft was sutured on the connective tissue of the occlusal side of the edentulous deepithelialized area, with a horizontal mattress suture and an interrupted suture, to secure the graft in place (Figure 5). Then, the second graft was harvested from the other side of the palate, which was of 20 mm length and 7 mm in width (Figure 6). This second graft was cut in two parts; the first part, which was about 13 mm in length, was sutured on the periosteum of the buccal side, with a horizontal mattress suture on the apical side and with a single interrupted suture on the first graft on the deepithelialized anatomical papillae (Figure 7).

**Figure 8:**
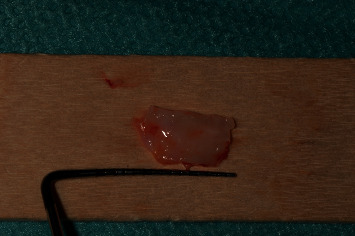
Second part of the second deepithelialized free gingival graft.

**Figure 9:**
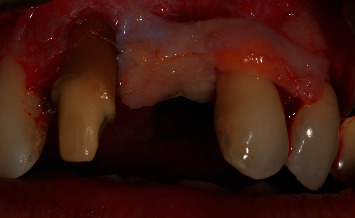
Sutured multilayered connective tissue graft (frontal view).

**Figure 10:**
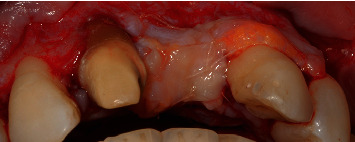
Sutured multilayered connective tissue graft (occlusal view).

After securing and stabilizing the first part of the second graft in place, the second part of the second graft, which was about 7 mm length (Figure 8), was then sutured occlusally with two sling sutures around the right maxillary central incisor and the left maxillary lateral incisor (Figures 9 and 10).

**Figure 11:**
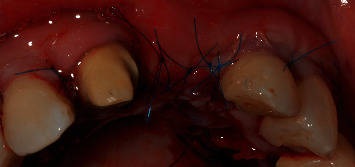
Suturing of the flap.

Coronal advancement of the buccal flap was obtained by means of two split-thickness incisions: a deep one, to cut the muscle insertions in the periosteum, and a superficial one, to detach the muscle inserting in the inner aspect of the mucosa lining the flap. This second incision allowed for the coronal advancement of the flap. The flap mobilization was considered appropriate when the buccal horizontal incision of the edentulous area reached the palatal horizontal incision without any tension. Sling sutures suspended around the palatal cingula of the involved teeth were used to anchor the surgical papillae to the corresponding deepithelialized anatomical papillae. Single interrupted sutures, with 6/0 prolene, were performed to have a complete closure between the horizontal incisions of the edentulous area and the palatal island flap (Figure 11).

### 3.2. Second Mucogingival Surgery

**Figure 12:**
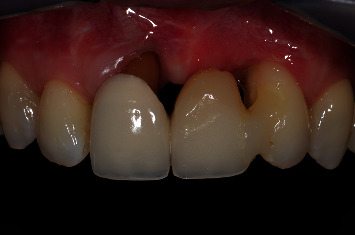
Healing after 6 months. Incomplete soft-tissue augmentation is evident.

**Figure 13:**
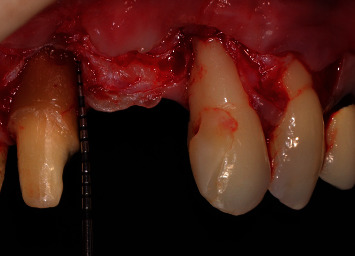
Probing depth after flap elevation.

**Figure 14:**
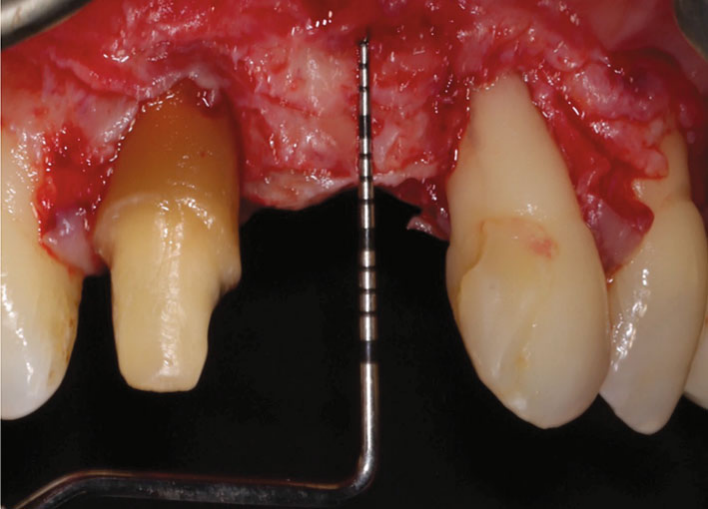
The previous connective tissue graft augmentation is evident after flap elevation.

Due to incomplete soft-tissue augmentation, we performed a second mucogingival surgery, 6 months after the first one (Figure 12). There was a complete absence of pocketing around the area of the first surgery. The same flap design, which was applied in the first surgery, was utilized. After split-thickness flap elevation, probing depth around the anterior maxillary teeth was 2 mm (Figures 13 and 14), which could mean that we might have a connective tissue attachment to the root surface of the right maxillary central incisor and the left maxillary lateral incisor.

The first graft, which was harvested from the right palate and was about 20 mm in length, was folded and then secured by two vertical mattress sutures, with 6/0 PGA sutures, to form a two-layered graft. The two-layered graft was sutured on the connective tissue of the occlusal side of the edentulous deepithelialized area with a horizontal mattress suture and an interrupted suture to secure the graft in place.

A second graft was then harvested from the other side of the palate and was of 25 mm length and 7 mm in width. This second graft was cut in two parts; the first part (about 18 mm length) was sutured on the periosteum of the buccal side, with a horizontal mattress suture on the apical side and with a single interrupted suture on the first graft and on the deepithelialized anatomical papillae. This graft, which was 5 mm longer than the buccal one done in the first surgery, covered the buccal area from the right maxillary central incisor up to the left maxillary lateral incisor.

**Figure 15:**
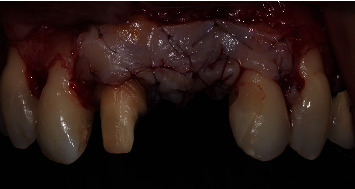
Sutured multilayered connective tissue graft (3 layers of occlusal connective tissue graft and one layer of buccal connective tissue graft).

**Figure 16:**
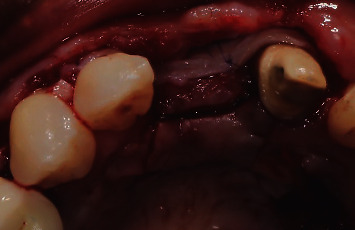
Connective tissue graft sutured on the buccal side. Coronal incision line of the palatal island flap is evident.

**Figure 17:**
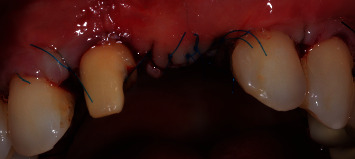
Final suturing of the flap with interrupted sutures.

**Figure 18:**
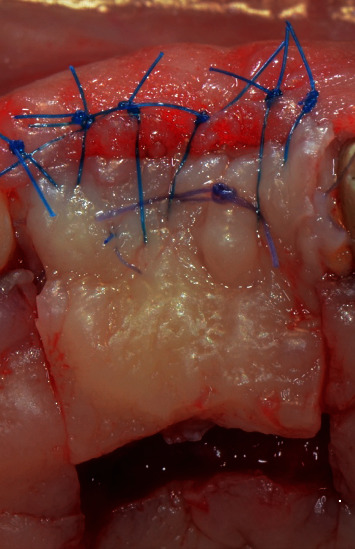
Primary intention closure with palatal island flap.

After the first part of the second graft was secured in place and was completely stable, the second part of the graft (which was about 7 mm length) was then sutured occlusally with two sling sutures around the right maxillary central incisor and the left maxillary lateral incisor (Figures 15 and 16). All the sutures used to stabilize the grafts in place were 6/0 PGA sutures. Flap closure was obtained in the same way as the first surgery by utilizing interrupted and sling sutures with 6/0 prolene (Figure 17). The palatal island flap facilitated primary intention closure of the flap as shown in Figure 18. In both surgeries, the palatal wound was protected with cyanoacrylate and collagen pack as previously described in literature [17], which helped limit the patient's postoperative discomfort, despite having multiple grafts harvested from both palatal sides.

### 3.3. Laser Frenectomy

We proposed the patient to undergo a strip technique [18, 19] to correct the amount of keratinized tissue and remove all muscular insertions. The patient was satisfied with the outcome and declined to undergo another graft harvesting surgery, so we performed a frenectomy with an apically positioned flap, using laser; six months after the second mucogingival surgery, the frenum was removed with a diode laser [20, 21] to relieve muscle attachment in the area.

### 3.4. Prosthetic Reconstruction

**Figure 19:**
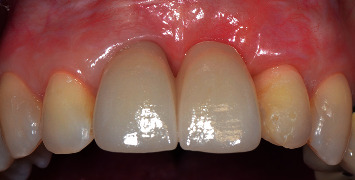
Final results, 16 months after the first mucogingival surgery.

**Figure 20:**
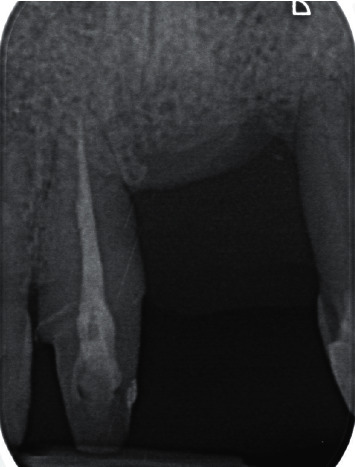
Final intraoral periapical radiograph.

A temporary cantilever resin-bonded bridge was used during the surgical periods to allow for proper tissue healing. Sixteen months after the first mucogingival surgery, the definitive prosthesis, a Zirconia cantilever bridge, restored the right and left maxillary central incisors, supported by a rest on the left maxillary lateral incisor. Clinical and radiographic final results can be seen in Figures 19 and 20. The patient was satisfied with the final results and aesthetic outcomes.

### 3.5. Overall Results of Postsurgical Follow-Ups

Probing around tissues was done 4 months postfrenectomy, which was 16 months after the first mucogingival surgery. The probing depth was 3 mm around the right maxillary central incisor. Buccal and palatal probing depths around the left maxillary lateral incisor were 5 mm. There were no signs of bleeding on probing. Gingival recessions were fully covered. Ten months after the second mucogingival surgery, a soft-tissue augmentation of 9 mm in the vertical direction was achieved. After the definitive prosthesis was placed, a satisfactory aesthetic outcome was reached. The color and shape of the bridge were in harmony with the other teeth (Figure 19), and the emergence profile of the pontic was easy to clean. The left maxillary lateral incisor showed no signs of mobility, while the right maxillary central incisor showed a degree I mobility.

## 4. Discussion

Vertical and horizontal bone augmentation procedures prior to dental implant placement employ different techniques to improve the overall bone volume and ensure final successful outcomes of dental implant therapy. Although vertical bone augmentation procedures, which include vertical GBR, onlay grafting, inlay grafting, and distraction osteogenesis [4, 5], are as technique sensitive as horizontal bone augmentation procedures, they are still more challenging. In fact, vertical bone augmentation procedures are associated with a high complication rate, mainly soft-tissue dehiscence and bone graft exposure [22], resulting in partial or complete loss of the bone-grafting material. In a systematic review on the use of nonresorbable membranes in vertical bone augmentation, the range of reported complications was 0%-45% [4].

In the present case, the patient previously underwent a vertical GBR procedure in the anterior maxilla, utilizing an e-PTFE membrane with an underlying autogenous/xenograft of a 1 : 1 ratio. In general, early or late exposure of membranes is considered as the main cause of GBR failure due to subsequent infection and contamination of the underlying bone grafting material [5, 23–25]. In a recent randomized controlled clinical trial, Cucchi et al. [26] reported a 15% healing complication rate in posterior mandibles treated with vertical bone augmentation utilizing nonresorbable e-PTFE membranes. Similarly, Simion et al. [27] experienced an 18% healing complication rate when the membrane technique was utilized with autogenous or allografts for vertical bone augmentation. The same research group reported similar findings when the e-PTFE membrane was utilized in combination with autograft/xenograft with a 1 : 1 ratio [28].

In the present case, GBR failure in the anterior maxilla is classified as “complete failure,” according to the classification proposed by Checchi et al. [29], which is strictly confined to the aesthetic consequences and treatment options of bone augmentation procedure failures in the atrophic anterior maxillae. In this classification, complete failure of bone augmentation procedures in the anterior maxilla is defined as “failure of the whole procedure which needs to be performed again, often with lower amount and poorer quality of hard and soft tissues than those present in the initial pre-surgical condition” [29]. However, since performing the same surgical procedure all over again might be unpredictable, a nonsurgical correction could be an alternative, which is usually influenced by the patient's decision [29]. In this scenario, the patient might not want to undergo a new bone augmentation procedure due to previous failure.

It must be noted that the correction of failed bone augmentation procedures in the anterior maxilla is more challenging than that in the mandible due to higher aesthetic demands [29]. Therefore, soft-tissue grafting is recommended in the reconstruction of a deficient alveolar ridge in the regions of high aesthetic demands [9]. In this context, different soft-tissue grafting techniques are applied in the correction of alveolar ridge defects for aesthetic purposes such as deepithelialized free gingival grafts [10], epithelial and subepithelial connective tissue grafts, roll pedicle graft, onlay graft, interpositional graft, and combined onlay interpositional graft [30–36], and the differences in the outcomes between different grafting procedures for deficient ridge reconstruction have been investigated. For example, Agarwal and Gupta [9] assessed the differences between subepithelial connective tissue graft in a pouch and combined onlay/connective tissue graft for the reconstruction of class III (Seibert) alveolar ridge defects. Findings of this study demonstrated that the combined onlay/connective tissue graft resulted in more significant gains in the horizontal and vertical ridge dimensions, after 3 and 6 months compared to baseline, due to better revascularization of the onlay graft and better control of the buccolingual and apicocoronal augmentation during the surgery [37].

In another randomized controlled clinical trial, vascularized interpositional periosteal-connective tissue graft (pediculated graft) and free subepithelial connective tissue graft were compared in correcting alveolar ridge deficiency of the anterior maxilla, however in Seibert class I [8]. The authors concluded that after 6 months, sites treated by the pediculated graft were superior in maintaining the initially augmented volume and showed less shrinkage of the graft, attributed to better perfusion of the pediculated graft [8].

Taking all the previous findings together, the GBR failure in the present case was corrected with soft-tissue grafting, taking into consideration the classification proposed by Checchi et al. [29] and also the patient's decision; the patient refused to undergo a new bone augmentation procedure because of the previous failure of the procedure. Due to the severe lack of soft-tissue volume in both the apicocoronal and buccolingual dimensions in the anterior edentulous maxillary area and the simultaneous gingival recession of adjacent teeth, GBR failure was corrected with “multiple soft tissue grafts” in two separate mucogingival surgeries, first to create an initial soft-tissue volume that would serve as a platform for successive soft-tissue augmentation to further increase soft-tissue volume in the edentulous area and second to cover the gingival recessions of adjacent teeth.

The surgical technique we used was similar to the subepithelial connective tissue graft (CTG) augmentation procedures, especially the one described by Zucchelli et al. [10], but with several modifications. In their novel subepithelial connective tissue graft technique for soft-tissue augmentation in a localized class III ridge defect (Seibert) in the anterior maxilla, Zucchelli et al. [10] utilized two folded subepithelial connective tissue grafts , obtained from depithelializing free gingival grafts, to create double thickness CTGs; one sutured buccolingually to create a platform and treat the horizontal component of the soft-tissue defect, and the second graft was sutured above the occlusal surface of the platform, to treat the vertical component of the soft-tissue defect. In the present case report, we have modified this technique.

First, we have reconstructed soft tissues in two separate mucogingival surgeries not only due to the larger defect area and the severely compromised soft-tissue quantity and quality following GBR failure but also due to the unpredictable shrinkage of augmented tissues [33]. Second, for the buccal flap design, we have adopted the one described for coronally advanced flap in the treatment of multiple gingival recessions [12, 13]; however, we have added two vertical releasing incisions, distal to the right and left maxillary canines, in order to better mobilize the flap because of the larger defect area, while Zucchelli et al. [10] avoided the incorporation of any vertical releasing incisions.

Third, we utilized the palatal island flap, as described by Tinti et al. [14] and modified by Hürzeler et al. [15], to move the palatal horizontal incision more coronally for about 3-4 mm to achieve primary intention closure.

Walter et al. [33] previously described a two-step approach for localized alveolar ridge augmentation of class III Seibert in the anterior maxilla, using different soft-tissue grafts: onlay-interpositional graft and subepithelial connective tissue graft. The first soft-tissue augmentation procedure was immediately followed by a separate frenulotomy; then three months later, a second soft-tissue surgical procedure was performed, with achieved satisfactory aesthetic outcomes. In the present case, we have also applied a two-step approach, but we have completed the two mucogingival surgeries first, with a 6-month interval between both surgeries, and then we performed frenectomy, 6 months after the second mucogingival surgery, keeping in mind that about 4-6 months are required for soft-tissue remodeling of the augmented tissues [33].

One of the main advantages of using soft-tissue grafting in correcting an alveolar ridge defect is that it is not as technique sensitive as bone augmentation procedures [38]. However, soft-tissue grafting techniques require a second surgical site, i.e., donor site to harvest the soft-tissue graft [38] and could be associated with unpredictable soft-tissue shrinkage over time, due to the long-term healing process [33], also around pontic areas.

Data on volumetric changes around pontic areas after soft-tissue augmentation procedures are available from retrospective 5-year [39] and 10-year [40] follow-up studies. Sanz-Martín et al. [39] reported that at 5 years, 24 patients receiving fixed dental prosthesis, with and without subepithelial CTGs, showed the same volumetric stability over the period of 5 years. In fact, the changes in soft-tissue pontic height amounted to a loss in the height of 0.34 mm in the group that received CTG and 0.35 mm in the group that did not receive any CTG, noting that there were no statistically significant differences in the baseline linear measurements between both groups [39]. Out of the 24 patients that were enrolled in the previous study, 17 were evaluated for a 10-year follow-up [40], with a calculation between the 5- and 10-year results being performed. Results showed that there were no statistically significant differences in pontic height between 5-year and 10-year results, suggesting a slight but continuous loss of tissue volume from baseline to 5 and 10 years, without any significant differences between sites with or without soft-tissue volume augmentation.

At the 16-month follow-up of the present case, no bleeding on probing was evident, gingival recessions were fully covered, and even levels of gingival margins were achieved. The soft tissues were stable. However, soft-tissue shrinkage, even if slight, is to be expected over time, also around pontics as confirmed by previous authors [39, 40].

## 5. Conclusions

The following conclusions can be inferred from the present case report:

1. Multilayered connective tissue grafting technique might be successful in correcting soft tissues after failed bone augmentation procedures
2. Vertical soft-tissue augmentation is possible with this technique
3. Two mucogingival surgeries were needed due to the large defect area
4. Palatal island flap is utilized with this technique to easily achieve a primary intention closure of the flap
5. Slight soft-tissue shrinkage is still to be expected over time
